# Comparative analysis of three fecal continence scoring systems in anorectal malformations: inter-score agreement and prognostic factors

**DOI:** 10.1007/s00383-026-06491-7

**Published:** 2026-06-21

**Authors:** Galib Bairamovi, Önder Özden, Hülya Binokay, Recep Tuncer

**Affiliations:** 1 Department of Pediatric Surgery, Faculty of Medicine, Baskent University, Adana, Turkey; 2 Department of Pediatric Surgery, Faculty of Medicine, Çukurova University, Adana, Turkey; 3 Department of Biostatistics, Faculty of Medicine, Çukurova University, Adana, Turkey

**Keywords:** Anorectal malformation, Fecal continence, Kelly score, Holschneider score, Templeton score, Agreement analysis

## Abstract

**Purpose:**

To evaluate inter-score correlation and categorical agreement among the Kelly, Holschneider, and Templeton fecal continence scores in patients with anorectal malformations (ARM), and to identify predictors of poor continence.

**Methods:**

In this retrospective single-center cohort, 110 surgically treated ARM patients (2007–2017) were assessed at latest follow-up using all three scores. Correlation was tested with Spearman analysis, categorical agreement with weighted kappa, and independent risk factors with multivariable logistic regression for each scoring system.

**Results:**

Correlations were moderate across all pairs (ρ = 0.52–0.68; p < 0.001). Agreement was substantial between Kelly–Holschneider (κ = 0.652) and Holschneider–Templeton (κ = 0.684), but moderate between Kelly–Templeton (κ = 0.595). Poor continence rates differed by instrument (Kelly 18.2%, Holschneider 10.9%, Templeton 17.3%). Independent predictors of poor continence varied by scoring system: weak anal tone and fecal incontinence for Kelly, urinary incontinence for Holschneider, and male sex and systemic pathology for Templeton.

**Conclusion:**

Although the three scores are correlated, clinically meaningful categorical discordance exists. Single-score assessment may therefore be misleading. Combined use of multiple validated continence scores with objective functional assessment is recommended for comprehensive ARM follow-up.

## Introduction

Anorectal malformations (ARM) are complex congenital anomalies with an estimated incidence ranging from 1 in 2,100 to 1 in 5,000 live births [1, 2], frequently occurring as part of the VACTERL association [3]. Although advances in surgical techniques such as posterior sagittal anorectoplasty (PSARP) have significantly improved anatomical reconstruction outcomes [4, 5], a substantial proportion of patients continue to experience functional problems, including fecal incontinence, constipation, and soiling [6, 7]. These issues adversely affect quality of life by impacting school performance, peer relationships, and self-esteem, and create significant emotional stress for parents [8, 9].

Various scoring systems have been developed to evaluate fecal continence in ARM patients. The Kelly score (0–6) focuses on sphincter function and soiling [10]. The Holschneider score (0–14) incorporates more comprehensive parameters, including stool consistency, rectal sensation, and gas–stool discrimination [11]. The Krickenbeck classification is among the most widely used scoring systems, evaluating voluntary bowel movements, soiling, constipation, and social problems [12]. The Templeton score (0–4.5) emphasizes social dimensions such as toilet training and social acceptability [13]. All of these scoring systems involve subjective assessment, as they rely on patient or parent reporting.

Despite their widespread use, limited data exist regarding inter-score agreement when these scoring systems are applied to the same patient cohort. Brisighelli et al. [14] compared the Holschneider, Rintala, and Krickenbeck scores but did not include the Kelly and Templeton scores. Shaari et al. [15] reported poor to moderate agreement (κ = 0.256–0.343) among different scores, while Mohamed et al. [16] highlighted validation deficiencies. To date, no study in the literature has simultaneously compared the Kelly, Holschneider, and Templeton scores in the same cohort, objectively measured inter-score agreement, and identified prognostic factors.

The objectives of this study were therefore twofold: first, to determine the correlation and categorical agreement among the Kelly, Holschneider, and Templeton continence scores when applied simultaneously to the same cohort of ARM patients; and second, to identify independent risk factors associated with poor continence as defined by each of the three scoring systems.

## Materials and methods

### Study design and ethics approval

This retrospective cohort study was conducted at a single tertiary pediatric surgery center and included all patients who underwent definitive surgical repair for anorectal malformations (ARM) between January 2007 and December 2017. This study protocol was approved by the Çukurova University Faculty of Medicine Ethics Committee on April 10, 2020 (Meeting No: 98, Decision No: N-5). Informed consent was obtained from the parents or legal guardians of all patients. This study adhered to the Strengthening the Reporting of Observational Studies in Epidemiology (STROBE) guidelines.

### Patient population

Inclusion criteria were: (1) confirmed diagnosis of ARM, (2) definitive surgical repair performed at our institution, (3) a minimum follow-up period of 3 years following the definitive procedure, and (4) completion of toilet training at the time of assessment (age ≥ 4 years). Exclusion criteria included: (1) early postoperative mortality, (2) patients with unclosed colostomy at final follow-up, and (3) incomplete medical records or follow-up data precluding accurate scoring.

**Fig. 1:**
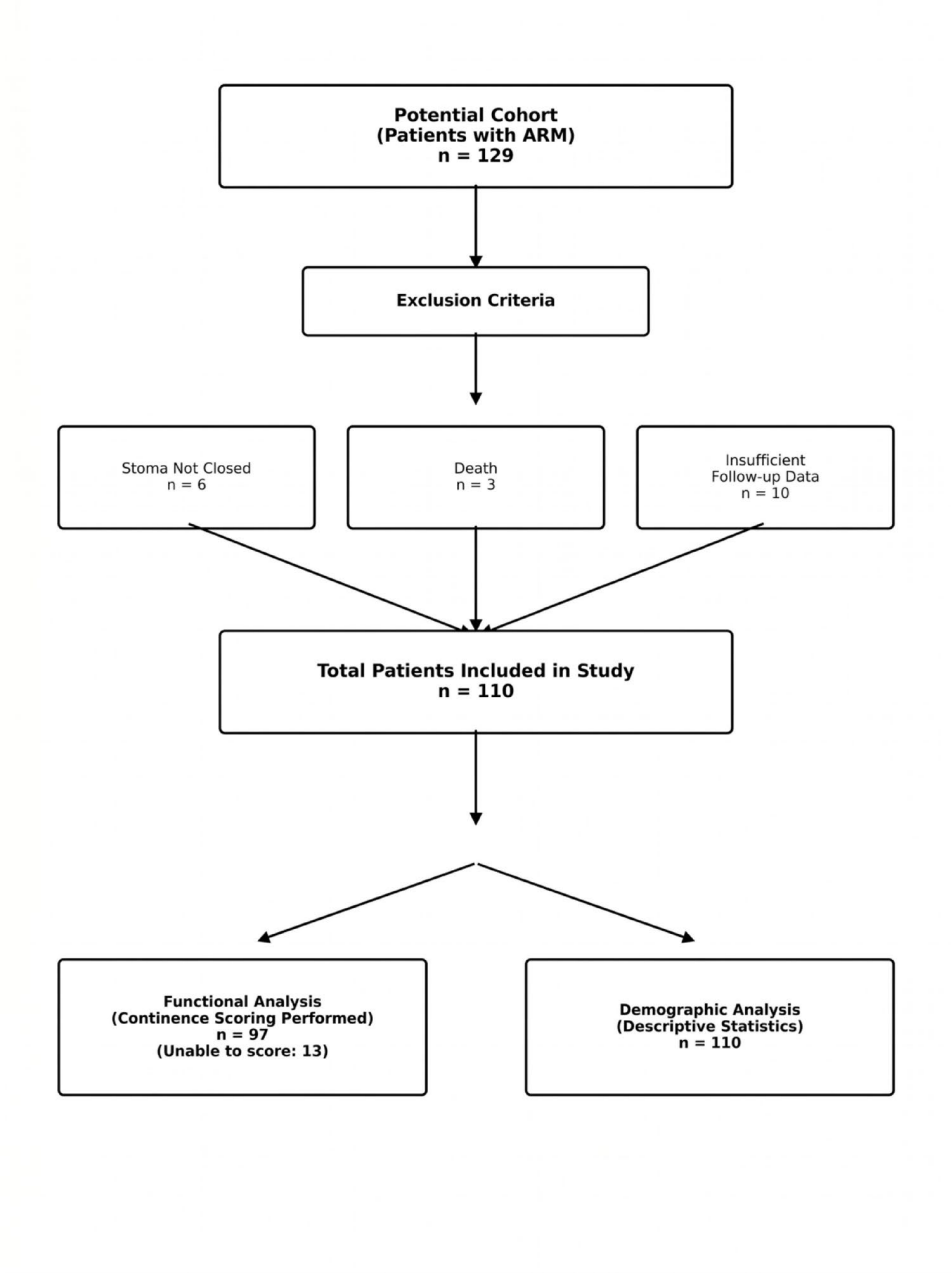
Patient flow diagram showing selection process from initial screening (n = 129) to final analysis cohort (n = 97 for continence scoring)

Patient selection is shown in Fig. 1. Initial screening identified 129 patients with potential ARM diagnosis. Nineteen patients were excluded: 6 unclosed colostomy, 3 who died in early postoperative period, and 10 with insufficient follow-up data. The study cohort comprised 110 ARM patients. Among these, 97 patients (88.2%) had complete data for all three continence scores and were included in the comparative functional analysis. Demographic analysis was performed on all 110 patients.

### Data collection

Medical records were retrospectively reviewed, and the following data were extracted: demographic characteristics (sex, age at definitive surgery, current age at follow-up), type of ARM according to the Wingspread classification, associated congenital anomalies (vertebral, cardiac, renal, limb anomalies), fistula localization, sacral anomalies, history of neurosurgical intervention (e.g., for tethered cord), urinary tract pathology, and details of surgical procedures.

**Urinary tract pathology** was defined as the presence of any structural or functional abnormality of the urinary system documented in medical records, including vesicoureteral reflux (VUR), neurogenic bladder, hydronephrosis, renal agenesis or dysplasia, and recurrent urinary tract infections requiring prophylactic antibiotics.

**Neurosurgical intervention** was included as a predictor variable, serving as a proxy indicator for tethered cord syndrome rather than as a direct pathophysiological determinant. This approach was chosen because tethered cord status was not systematically documented in all patients’ medical records, whereas neurosurgical procedures (primarily tethered cord release) were consistently recorded.

### Continence scoring systems

Fecal continence was assessed simultaneously using three validated scoring systems during the most recent follow-up visit (minimum 3 years post-repair):

**Kelly Clinical Continence Score (0–6):** Evaluates three parameters: (1) degree of soiling/staining, (2) frequency of accidents, and (3) need for a bowel management program. Scores of 5–6 are classified as good, 3–4 as fair, and 0–2 as poor continence [10].

**Holschneider Score (0–14):** A comprehensive score incorporating clinical and functional parameters including frequency and severity of soiling, ability to hold stool, need for dietary or medical management, sensation, and manometric findings. Scores of 10–14 are classified as good, 5–9 as fair, and 0–4 as poor continence [11].

**Templeton Score (0–4.5):** Emphasizes social dimensions of continence, evaluating frequency of fecal accidents and impact on social functioning. Scores of 4–4.5 are classified as good, 2–3.5 as fair, and 0–1.5 as poor continence [13].

All scores were calculated by two independent assessors, and discrepancies were resolved by consensus with a senior pediatric surgeon.

### Statistical analysis

Statistical analyses were performed using SPSS version 25.0 (IBM Corporation, Armonk, NY, USA) and R Studio (Version 4.2.2). Continuous variables were expressed as mean ± standard deviation (SD) or median with interquartile range (IQR), depending on the normality of distribution as assessed by the Shapiro–Wilk test. Categorical variables were expressed as frequencies and percentages.

Correlation between the three continence scores was assessed using Spearman’s rank correlation coefficient (ρ). Categorical agreement between score pairs was evaluated using weighted Cohen’s kappa (κ) statistic with 95% confidence intervals (CI). Kappa values were interpreted as follows: < 0.20 = poor, 0.21–0.40 = fair, 0.41–0.60 = moderate, 0.61–0.80 = good (substantial), and 0.81–1.00 = excellent (almost perfect) agreement [17].

The categorical distributions of the three scores (good/fair/poor) were compared using the chi-square test or Fisher’s exact test, as appropriate.

To identify independent predictors of poor continence, separate multivariate binary logistic regression models were constructed for each of the three scores. The dependent variable was poor continence (yes/no) as defined by each respective score. Independent variables included in the models were selected based on clinical relevance and a univariate screening threshold of p < 0.10. Firth bias-reduced logistic regression was applied to account for rare events. Backward stepwise variable selection was used; variables with p < 0.05 retained in final models. Results were expressed as adjusted odds ratios (aOR) with 95% CIs. Model fit was evaluated using Hosmer–Lemeshow goodness-of-fit test, and model discrimination using Nagelkerke R^2^.

Statistical significance was set at p < 0.05 (two-tailed).

## Results

### Patient demographics and clinical characteristics

The study cohort comprised 110 patients (59 males [53.6%] and 51 females [46.4%]). The age distribution was as follows: 0–5 years (19.1%), 5–10 years (50.9%), and > 10 years (30.0%).

According to the Wingspread classification, intermediate-type ARM was the most common malformation (n = 44, 40.0%), followed by low-type (n = 32, 29.1%), high-type (n = 22, 20.0%), and cloacal anomalies (n = 6, 5.5%). Six patients (5.5%) had undergone prior surgery at outside institutions and were referred for reoperation.

Regarding fistula localization, rectoperineal fistula was the most common anatomical variant (29.1%), followed by rectovestibular (20.9%), rectourethral (19.1%), and atresia without fistula (16.4%). Rectovesical fistula (3.6%) and cloacal anomalies (5.5%) were less common.

Associated congenital anomalies were frequently observed. Urinary system anomalies were the most common associated pathology (n = 48, 43.6%), including vesicoureteral reflux, renal agenesis, neurogenic bladder, and hypospadias. Vertebral anomalies were identified in 22 patients (20.0%), including tethered cord, sacral agenesis, and vertebral fusion defects. Gastrointestinal anomalies (particularly esophageal atresia) were present in 22 patients (20.0%), and other systemic pathologies in 25 patients (22.7%) (Table 1).

**Table 1:** Patient Demographics and Clinical Characteristics (N = 110)

Variable	n	%
Sex		
Female	51	46.4
Male	59	53.6
Age group		
0–5 years	21	19.1
5–10 years	56	50.9
> 10 years	33	30.0
Wingspread classification		
High-type	22	20.0
Intermediate-type	44	40.0
Low-type	32	29.1
Cloaca	6	5.5
Prior surgery at outside institution	6	5.5
Fistula type		
Rectoperineal	32	29.1
Rectovestibular	23	20.9
Rectourethral	21	19.1
No fistula	18	16.4
Cloacal	6	5.5
Rectovesical	4	3.6
Unknown (prior surgery at outside institution)	6	5.5
Associated anomalies		
Urinary system	48	43.6
Vertebral	22	20.0
Gastrointestinal	22	20.0
Other systemic	25	22.7

### Surgical management and complications

Initial management consisted of colostomy in 64 patients (58.2%), anal dilatation in 33 (30.0%), anoplasty in 8 (7.3%), and primary anorectoplasty in 5 (4.5%). Definitive surgical repair was performed using posterior sagittal anorectoplasty (PSARP) in 88 patients (80.0%), anoplasty in 12 (10.9%), anterior sagittal anorectoplasty (ASARP) in 5 (4.6%), laparoscopic-assisted anorectoplasty (LARP) in 2 (1.8%), and PSARVUP in 3 (2.7%).

Reoperation was required in 20 patients (18.2%). Indications for reoperation included fecal incontinence (n = 8), anal stenosis (n = 6), mislocated neo-anus (n = 4), and rectal prolapse (n = 1). Four patients with persistent incontinence underwent an antegrade continence enema (ACE) procedure.

Postoperative complications were classified according to the Clavien-Dindo system [18] (Table 2). Early postoperative complications were observed in 23 patients (20.9%), with wound infection (12.7%) and sepsis (6.4%) being the most common. Late complications occurred in 37 patients (33.6%), including rectal prolapse (13.6%), fecal incontinence (12.7%), and anal stenosis (5.4%). The majority of patients with complications (92.7%) experienced Grade I complications requiring no intervention, while severe complications (Grade III–IV) were observed in 4.5% of cases.

**Table 2:** Postoperative Complications According to Clavien-Dindo Classification

Complication	n	%
Early Complications		
None	87	79.1
Wound infection	14	12.7
Sepsis	7	6.4
Recurrent fistula	1	0.9
Globe vesicale	1	0.9
Late Complications		
None	73	66.4
Rectal prolapse	15	13.6
Fecal incontinence	14	12.7
Anal stenosis	6	5.4
Other	2	1.8
Clavien-Dindo Grade		
Grade I	102	92.7
Grade II	3	2.7
Grade III	3	2.7
Grade IV	2	1.8
Grade V	0	0.0

### Continence score distributions and comparison

Continence assessment was performed in 97 patients (88.2%) using the Kelly, Holschneider, and Templeton scoring systems; 13 patients (11.8%) were lost to follow-up. Median scores were 5 (range 0–6) for Kelly and 13 (range 2–14) for Holschneider. The mean Templeton score was 3.73 ± 1.60, with a median of 4.5 (range 0.5–4.5).

Categorical continence distributions differed significantly among the three scoring systems (p = 0.027), with poor continence rates of 18.2% (Kelly), 10.9% (Holschneider), and 17.3% (Templeton) (Table 3). The discordance was particularly evident in the fair continence category: Kelly classified 22.8% as fair, whereas Templeton classified only 9.9% in this category.

**Table 3:** Categorical Distribution of Continence Scores (N = 97)

Continence Category	Kelly, n (%)	Holschneider, n (%)	Templeton, n (%)
Good	52 (47.3)	65 (59.1)	67 (60.9)
Fair	25 (22.8)	20 (18.1)	11 (9.9)
Poor	20 (18.2)	12 (10.9)	19 (17.3)
Median (range)	5 (0–6)	13 (2–14)	4.5 (0.5–4.5)*

Of 110 total patients, 97 (88.2%) completed continence assessment; 13 (11.8%) were lost to follow-up and excluded from scoring analyses

^*^Mean Templeton score: 3.73 ± 1.60

Kelly: good 5–6, fair 3–4, poor 0–2. Holschneider: good 10–14, fair 5–9, poor 0–4. Templeton: good 4–4.5, fair 2–3.5, poor 0–1.5

### Correlation and agreement between scoring systems

Spearman analyses revealed moderate correlations among all score pairs (ρ = 0.52–0.68; all p < 0.001). Categorical agreement was evaluated using weighted kappa analysis (Table 4). Good (substantial) agreement was found between Kelly and Holschneider (κ = 0.652; 95% CI: 0.521–0.783; p < 0.001) and between Holschneider and Templeton (κ = 0.684; 95% CI: 0.547–0.821; p < 0.001). Moderate agreement was observed between Kelly and Templeton (κ = 0.595; 95% CI: 0.458–0.732; p < 0.001).

**Table 4:** Correlation and Categorical Agreement Between Continence Score Pairs

Score Pair	Spearman ρ	p value	Weighted κ (95% CI)	p value	Agreement Level
Kelly–Holschneider	0.68	< 0.001	0.652 (0.521–0.783)	< 0.001	Good (substantial)
Holschneider–Templeton	0.64	< 0.001	0.684 (0.547–0.821)	< 0.001	Good (substantial)
Kelly–Templeton	0.52	< 0.001	0.595 (0.458–0.732)	< 0.001	Moderate

Interpretation of κ: < 0.20 poor; 0.21–0.40 fair; 0.41–0.60 moderate; 0.61–0.80 good (substantial); 0.81–1.00 excellent (almost perfect)

### Factors associated with poor continence (Univariate Analysis)

Univariate analysis identified several factors significantly associated with poor continence across the three scoring systems (Table 5).

**Table 5:** Univariate Analysis: Factors Associated with Poor Continence

Factor	Kelly Score (mean ± SD)	p	Holschneider Score (mean ± SD)	p	Templeton Score (mean ± SD)	p
Neurosurgical intervention		0.001		< 0.001		0.008
Yes	1.9 ± 1.6		6.2 ± 3.0		2.4 ± 1.3	
No	4.2 ± 1.9		11.0 ± 3.5		3.9 ± 1.6	
Urinary tract pathology		0.031		0.019		0.034
Yes	3.4 ± 2.1		9.2 ± 3.9		3.3 ± 1.7	
No	4.5 ± 1.7		11.7 ± 3.2		4.1 ± 1.4	
Vertebral pathology		0.098		0.022		0.128
Yes	3.5 ± 2.3		9.5 ± 4.3		3.2 ± 1.8	
No	4.1 ± 1.9		10.9 ± 3.5		3.9 ± 1.5	
Sex		0.479		0.372		0.023
Female	4.4 ± 1.9		11.1 ± 3.7		4.1 ± 1.4	
Male	3.6 ± 1.9		10.1 ± 3.7		3.4 ± 1.7	

### Independent predictors of poor continence (Multivariable Analysis)

Separate multivariable logistic regression models constructed for each scoring system further highlighted the differential sensitivity of the scores (Table 6).

**Table 6:** Multivariable Logistic Regression: Independent Predictors of Poor Continence

Scoring System	Predictor	aOR	95% CI	p value
Kelly Score	Weak anal tone	8.35	2.32–8.80	0.024
	Fecal incontinence	7.14	1.83–9.45	0.013
	Nagelkerke R^2^ = 0.62; Hosmer–Lemeshow p = 0.78	Nagelkerke R^2^ = 0.62; Hosmer–Lemeshow p = 0.78	Nagelkerke R^2^ = 0.62; Hosmer–Lemeshow p = 0.78	Nagelkerke R^2^ = 0.62; Hosmer–Lemeshow p = 0.78
Holschneider Score	Urinary incontinence	10.00	3.35–11.00	0.006
	Nagelkerke R^2^ = 0.57; Hosmer–Lemeshow p = 0.96	Nagelkerke R^2^ = 0.57; Hosmer–Lemeshow p = 0.96	Nagelkerke R^2^ = 0.57; Hosmer–Lemeshow p = 0.96	Nagelkerke R^2^ = 0.57; Hosmer–Lemeshow p = 0.96
Templeton Score	Male sex	11.16	3.32–13.65	0.027
	Systemic pathology	10.42	1.74–11.44	0.010
	Weak anal tone	8.01	1.69–10.03	0.009
	Nagelkerke R^2^ = 0.59; Hosmer–Lemeshow p = 0.70	Nagelkerke R^2^ = 0.59; Hosmer–Lemeshow p = 0.70	Nagelkerke R^2^ = 0.59; Hosmer–Lemeshow p = 0.70	Nagelkerke R^2^ = 0.59; Hosmer–Lemeshow p = 0.70

*aOR* adjusted odds ratio, *CI* confidence interval

Firth bias-reduced logistic regression was used to account for rare events

Backward stepwise variable selection was applied; variables with p < 0.05 retained in final models

## Discussion

This study represents one of the most comprehensive single-center analyses to date that simultaneously evaluates the correlation and categorical agreement among three widely used continence scoring systems (Kelly, Holschneider, and Templeton) in ARM patients, while identifying independent risk factors associated with poor continence. Our principal finding is that although moderate correlation exists among the three scores (ρ = 0.52–0.68), categorical agreement varies substantially across score pairs. Good (substantial) agreement was observed between Kelly and Holschneider (κ = 0.652) and between Holschneider and Templeton (κ = 0.684), whereas moderate agreement was found between Kelly and Templeton (κ = 0.595). These findings support our hypothesis that the three scores reflect different dimensions of continence.

The distribution of anatomic types among our 110 ARM patients is generally consistent with large cohorts reported in the literature [19, 20]. As expected, patients with high-type ARM, cloacal anomalies, and associated vertebral or sacral anomalies demonstrated significantly poorer fecal continence outcomes. These findings reaffirm the determinative role of malformation type, sacral-spinal integrity, and associated major anomalies on long-term functional outcomes.

### Agreement between scoring systems

One of the most striking findings of this study is the differential agreement levels observed among the three scoring systems. Our kappa analysis revealed varying degrees of agreement across score pairs. Good (substantial) agreement was found between Kelly and Holschneider (κ = 0.652; 95% CI: 0.521–0.783) and between Holschneider and Templeton (κ = 0.684; 95% CI: 0.547–0.821), while moderate agreement was observed between Kelly and Templeton (κ = 0.595; 95% CI: 0.458–0.732).

These findings suggest that Kelly and Templeton scores particularly focus on different dimensions of continence. The Kelly score primarily evaluates physiological parameters such as sphincter function and soiling [10], whereas the Templeton score emphasizes social dimensions including toilet training, social acceptability, and protective garment use [13]. The Holschneider score demonstrates good agreement with both scores because it offers a broader assessment encompassing both physiological (sphincter function, soiling) and functional (rectal sensation, gas-stool discrimination) parameters [11]. We specifically selected Kelly, Holschneider, and Templeton to represent distinct evaluation domains—physiological sphincter function, comprehensive functional parameters, and social quality-of-life dimensions—thereby capturing complementary rather than overlapping constructs. This combination allows assessment of whether a single-score approach is sufficient or whether multi-dimensional evaluation is necessary for comprehensive ARM continence assessment.

Similar findings exist in the literature. Brisighelli et al. [14] compared Holschneider, Rintala, and Krickenbeck scores in a series of 80 patients and reported varying differences among scores depending on ARM type. Shaari et al. [15] found poor to moderate agreement (κ = 0.256–0.343) among Kelly, Holschneider, and Krickenbeck scores and emphasized that no single score is sufficient alone. The higher agreement values observed in our study (κ = 0.595–0.684) may be attributed to differences in patient population or inter-rater agreement. In their recent comprehensive review, Mohamed et al. [16] highlighted validation deficiencies of older scoring systems and emphasized the importance of multidisciplinary assessments integrating quality-of-life measures.

Despite finding good agreement in kappa analysis, marked discordance was observed in the categorical classification (good/fair/poor) of the scores. Poor continence rates were 18.2% (Kelly), 17.3% (Templeton), and 10.9% (Holschneider). The discrepancy was even more pronounced in the fair continence category: Kelly classified 22.8% as fair, whereas Templeton classified only 9.9% in this category. This may result from the scores using different threshold values, as well as each score emphasizing a different aspect of continence. The practical implication is clear: a patient classified as having “good” continence by one system may be categorized as “fair” or “poor” by another system. This discordance creates uncertainty in clinical decision-making and complicates cross-study comparisons.

### The appendicitis analogy: the need for objective assessment

A useful clinical analogy can be drawn from the diagnosis of acute appendicitis. Although the Alvarado and AIR score is valuable, it has proven insufficient when used alone and is now routinely supplemented with objective imaging modalities [21]. Similarly, exclusive reliance on subjective clinical scores in ARM follow-up may not fully reflect true physiological capacity. The common limitation of all three scoring systems is their dependence on patient or parent reporting, which introduces inherent subjectivity and potential recall bias [22, 23].

This limitation points to the indispensable role of objective assessment modalities. Pelvic and spinal magnetic resonance imaging (MRI) provides critical structural information regarding sphincter complex anatomy, levator ani integrity, and neural abnormalities [25, 26]. In our series, MRI was performed in 36 patients, with pathological findings identified in 27 (24.5%). The strong relationship between pelvic MRI findings and the Holschneider score in our study suggests that this score may be more sensitive to complex anatomical variants and neurological deficits.

Voiding cystourethrography (VCUG) was performed in 52 patients (47.3%), revealing pathological findings in 24 (21.8%); this rate is consistent with the 20–50% range reported in the literature [24, 25]. Anorectal manometry measures resting and squeeze pressures, rectal compliance, and rectoanal reflexes [27]. Recent studies using high-resolution three-dimensional anorectal manometry have demonstrated its value in detailed sphincter assessment [28]. In our series, patients with abnormal intraoperative electrical stimulator findings demonstrated significantly lower postoperative continence scores across all three systems. This finding supports the routine use of intraoperative electrical stimulation not only for precise neo-anus positioning during surgery, but also as a prognostic indicator for counseling families regarding expected continence outcomes [29, 30].

### Risk factors and clinical implications

Our analysis identified several factors universally associated with poor continence across all three scoring systems. Patients with spinal pathology requiring neurosurgical intervention (tethered cord, sacral agenesis) had significantly lower continence scores across all systems (Kelly: 1.9 ± 1.6 vs 4.2 ± 1.9, p = 0.001; Holschneider: 6.2 ± 3.0 vs 11.0 ± 3.5, p < 0.001; Templeton: 2.4 ± 1.3 vs 3.9 ± 1.6, p = 0.008). In our series, 9 patients (8.2%) required neurosurgical intervention, a rate consistent with the 4–20% range reported in the literature [22, 29]. This finding underscores the importance of systematic spinal screening and early neurosurgical consultation in ARM patients.

Urinary tract pathology similarly demonstrated consistent adverse effects across all instruments. The presence of urinary anomalies was significantly associated with lower scores across all three systems (Kelly: 3.4 ± 2.1 vs 4.5 ± 1.7, p = 0.031; Holschneider: 9.2 ± 3.9 vs 11.7 ± 3.2, p = 0.019; Templeton: 3.3 ± 1.7 vs 4.1 ± 1.4, p = 0.034). Fistula localization, particularly rectovesical and high-type fistulas, and initial management requiring colostomy were universally associated with poorer outcomes.

Vertebral pathology was present in 22 patients (20.0%) in our series; this rate is lower than the 35–46% reported in some literature [30, 31]. Nevertheless, vertebral anomalies showed significant association with lower Holschneider scores (p = 0.022), highlighting the score’s sensitivity to anatomical factors affecting pelvic floor innervation. This finding suggests that the Holschneider score may be more sensitive to neurological deficits than other scores.

Importantly, our multivariable analysis revealed that each scoring system identified entirely different independent predictors of poor continence: weak anal tone and fecal incontinence for Kelly, urinary incontinence alone for Holschneider, and male sex, systemic pathology, and weak anal tone for Templeton. This differential sensitivity has not been reported previously and demonstrates that each score captures a unique aspect of continence dysfunction, with practical implications for score selection based on the specific research question or clinical context. Our use of Firth bias-reduced logistic regression provides robust quantitative estimates (aOR = 7.14–11.16) that account for rare events and inform clinical counseling regarding individual risk profiles.

The documented impact of urospinal comorbidities on ARM continence outcomes is consistent with extensive prior evidence [32–35]. Tethered cord and lumbosacral anomalies compromise pelvic floor innervation, producing dysfunction that may be resistant to surgical technique optimization alone [25, 31, 36]. Our findings strongly support systematic early investigation of these comorbidities in every ARM patient.

In contrast, perioperative factors including bowel management program implementation, anal dilatation duration, and postoperative complication severity did not show consistent association with long-term continence scores. This pattern suggests that ultimate continence prognosis is fundamentally determined by preoperative anatomic and neurological substrate rather than perioperative management variations. However, these interventions remain critical for optimizing patient outcomes within their existing capacities. The lower rate of postoperative constipation in our series (14.5% chronic) compared to the 30–80% reported in the literature demonstrates the importance of close outpatient follow-up and timely implementation of bowel management programs [37, 38].

### Proposed integrated assessment algorithm

**Fig. 2:**
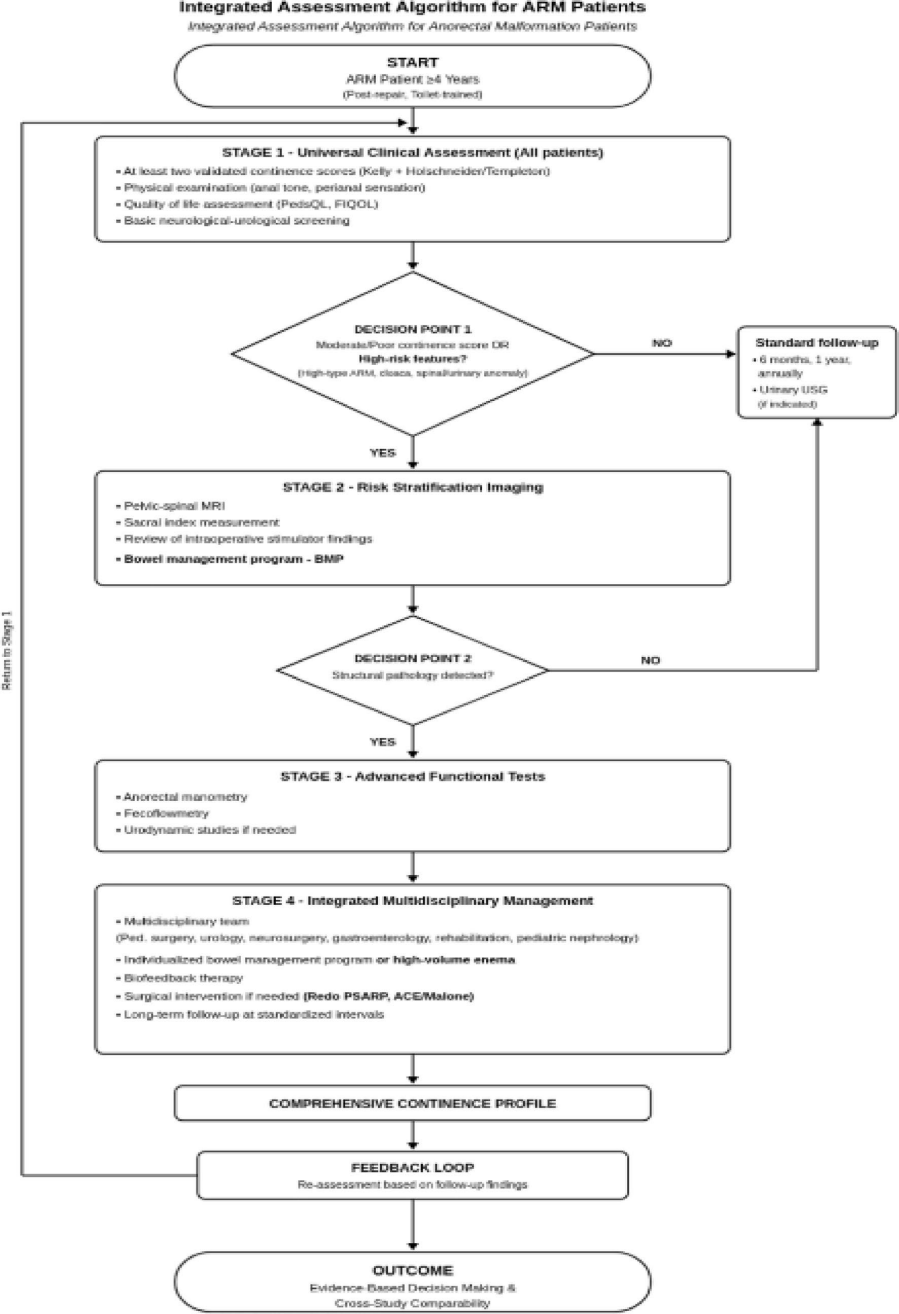
Proposed integrated multimodal assessment algorithm for fecal continence in anorectal malformation patients

The complementary role of objective functional and radiological investigations forms the basis of our proposed “Integrated Postoperative Assessment Algorithm for ARM.” This algorithm directly addresses the central clinical implication of our findings: that no single continence score is sufficient for comprehensive ARM assessment, as evidenced by our finding of categorical discordance (poor continence rates: Kelly 18.2%, Holschneider 10.9%, Templeton 17.3%) and score-specific differential sensitivity to distinct risk factors. Based on these findings, we propose a staged integrated assessment algorithm (Fig. 2) that optimizes resource utilization while ensuring comprehensive evaluation:

**Stage 1**—**Universal Clinical Assessment:** All ARM patients reaching postoperative age ≥ 4 years should undergo comprehensive clinical evaluation: (a) simultaneous application of at least two validated continence scores (we recommend Kelly plus Holschneider or Templeton to capture both physiological and social dimensions); (b) physical examination with anal tone assessment; (c) quality-of-life assessment using age-appropriate instruments; (d) basic neurological and urological screening.

**Stage 2**—**Risk-Stratified Imaging:** In patients with fair or poor continence scores or those with high-risk features (high-type ARM, cloacal anomalies, known spinal anomalies, urinary symptoms): (a) pelvic and spinal MRI; (b) sacral index measurement on radiographs; (c) review of intraoperative electrical stimulator findings from primary surgery.

**Stage 3**—**Advanced Functional Testing:** When Stage 2 identifies structural pathology: (a) anorectal manometry; (b) fecoflowmetry; (c) urodynamic studies if urinary symptoms are present.

**Stage 4**—**Integrated Multidisciplinary Management:** Individualized management plan by a multidisciplinary team including pediatric surgery, pediatric urology, pediatric neurosurgery, pediatric nephrology, pediatric gastroenterology, and rehabilitation specialists: (a) individualized bowel management programs; (b) biofeedback therapy; (c) planning for neurosurgical or urological intervention; (d) redo surgery assessment when anatomical correction is feasible.

This tiered approach optimizes resource utilization by reserving expensive and invasive investigations for patients most likely to benefit, while ensuring that high-risk patients receive comprehensive evaluation. The emphasis on integration—combining subjective scores with objective measures—addresses the inherent limitations of any single assessment modality.

### Strengths, limitations, and future directions

This study has several important strengths. Our cohort of 110 patients represents one of the largest single-center series simultaneously comparing multiple continence scoring systems. The use of multivariable analysis independently for each scoring system provides robust evidence of differential risk factor sensitivity. High inter-rater agreement (κ = 0.85) supports data reliability. The involvement of four different surgeons enhances generalizability beyond single-surgeon technical variations. Additionally, this is among the first studies to apply the Clavien-Dindo classification for systematic grading of postoperative complications in ARM patients, providing a standardized framework for complication reporting that facilitates cross-study comparison.

However, several limitations must be acknowledged. The retrospective design introduces potential selection and information bias. As a single-center study, findings may not be fully generalizable to other populations with different referral patterns or surgical practices. Thirteen patients (11.8%) could not be reached for follow-up scoring; however, their demographic and surgical characteristics did not differ significantly from the evaluated cohort, minimizing the likelihood of attrition bias. Additionally, continence scores are inherently subjective, and parent-reported data for young children may contain recall bias. In our cohort, 69.1% of patients were aged 5 years or older at assessment, an age at which parental recall is generally considered reliable for bowel function patterns; nevertheless, future studies incorporating objective metrics (anorectal manometry, fecoflowmetry) and validated quality-of-life instruments with psychometric validation would further strengthen assessment reliability.

Future research should prioritize prospective validation of integrated assessment algorithms in multi-center settings. Studies comparing the predictive validity of clinical scores versus objective tests for patient-reported outcomes (quality of life, social participation) are needed. Cost-effectiveness analyses of tiered assessment protocols will inform resource allocation. Long-term studies following patients into adulthood will clarify whether childhood continence patterns predict adult functional outcomes [39, 40]. Finally, the development and validation of composite scores incorporating both subjective and objective measures may offer a more holistic assessment tool for ARM patients.

## Conclusion

This comprehensive single-center study demonstrates that although the Kelly, Holschneider, and Templeton continence scores show moderate correlation (ρ = 0.52–0.68) and variable categorical agreement (κ = 0.595–0.684), clinically meaningful categorical discordance exists, with poor continence rates of 18.2%, 10.9%, and 17.3% respectively. These findings indicate that each score reflects a distinct dimension of continence—Kelly capturing physiological sphincter function, Holschneider representing comprehensive functional parameters, and Templeton emphasizing social quality of life. Single-score assessment may therefore be misleading, and no individual scoring system is sufficient for comprehensive ARM evaluation.

Our multivariable analysis revealed that each scoring system identified entirely different independent predictors of poor continence: weak anal tone and fecal incontinence for Kelly, urinary incontinence for Holschneider, and male sex and systemic pathology for Templeton. Spinal pathology requiring neurosurgical intervention (8.2% of our cohort) and urinary tract pathology (43.6%) emerged as consistent adverse factors across all systems, underscoring the critical importance of systematic early screening and multidisciplinary consultation for these comorbidities.

We recommend that postoperative ARM follow-up employ an integrated, risk-stratified algorithm combining at least two validated continence scores from different domains with objective assessment modalities (MRI, anorectal manometry, fecoflowmetry). Prospective, multi-center validation of this integrated approach is essential to advance evidence-based continence care and improve long-term quality-of-life outcomes in children with anorectal malformations.

## Data Availability

The datasets generated and/or analyzed during the current study are available from the corresponding author on reasonable request.
